# Signatures of Pleiotropy, Economy and Convergent Evolution in a Domain-Resolved Map of Human–Virus Protein–Protein Interaction Networks

**DOI:** 10.1371/journal.ppat.1003778

**Published:** 2013-12-05

**Authors:** Sara Garamszegi, Eric A. Franzosa, Yu Xia

**Affiliations:** 1 Bioinformatics Program, Boston University, Boston, Massachusetts, United States of America; 2 Department of Biostatistics, Harvard School of Public Health, Boston, Massachusetts, United States of America; 3 Department of Bioengineering, Faculty of Engineering, McGill University, Montreal, Quebec, Canada; University of Texas at Austin, United States of America

## Abstract

A central challenge in host-pathogen systems biology is the elucidation of general, systems-level principles that distinguish host-pathogen interactions from within-host interactions. Current analyses of host-pathogen and within-host protein-protein interaction networks are largely limited by their resolution, treating proteins as nodes and interactions as edges. Here, we construct a domain-resolved map of human-virus and within-human protein-protein interaction networks by annotating protein interactions with high-coverage, high-accuracy, domain-centric interaction mechanisms: (1) domain-domain interactions, in which a domain in one protein binds to a domain in a second protein, and (2) domain-motif interactions, in which a domain in one protein binds to a short, linear peptide motif in a second protein. Analysis of these domain-resolved networks reveals, for the first time, significant mechanistic differences between virus-human and within-human interactions at the resolution of single domains. While human proteins tend to compete with each other for domain binding sites by means of sequence similarity, viral proteins tend to compete with human proteins for domain binding sites in the absence of sequence similarity. Independent of their previously established preference for targeting human protein hubs, viral proteins also preferentially target human proteins containing linear motif-binding domains. Compared to human proteins, viral proteins participate in more domain-motif interactions, target more unique linear motif-binding domains per residue, and contain more unique linear motifs per residue. Together, these results suggest that viruses surmount genome size constraints by convergently evolving multiple short linear motifs in order to effectively mimic, hijack, and manipulate complex host processes for their survival. Our domain-resolved analyses reveal unique signatures of pleiotropy, economy, and convergent evolution in viral-host interactions that are otherwise hidden in the traditional binary network, highlighting the power and necessity of high-resolution approaches in host-pathogen systems biology.

## Author Summary

The goal of host-pathogen systems biology is to examine the complex interactions between species, such as those between a virus and its host. Analysis of protein-protein interaction (PPI) networks can identify general principles that distinguish between within-species and between-species interactions. However, PPI data are limited by their low resolution, and cannot provide detailed information about the physical mechanisms underlying interactions between proteins. Using protein domain-based annotation methods, we have constructed an integrated human-virus PPI network which better highlights the mechanistic differences between human-human and human-virus PPIs. Our findings suggest that viral proteins use unique strategies to interact with human proteins, a finding with significant implications for pathogen research.

## Introduction

Protein-protein interactions (PPIs) can be broadly classified into two fundamentally different classes: those within the same species, such as within-host PPIs, and those between different species, such as host-pathogen PPIs. Are there general, systems-level principles that distinguish host-pathogen PPIs (*exogenous interactions*) from within-host PPIs (*endogenous interactions*)? Surprisingly, little is known about the existence and nature of such global principles, in part because they are not amenable to investigation by traditional methods, which examine specific host-pathogen PPIs individually. The most well-studied host-pathogen interaction systems are host-virus interactions, and the combined results of decades of detailed studies on specific host-virus interactions suggest that such global principles may exist. Endogenous interactions among host proteins are expected to be cooperative: proteins encoded within the same genome interact with one another to carry out biological function in a coordinated and synergistic fashion. On the contrary, exogenous interactions between viral proteins and host proteins are expected to be largely antagonistic: viruses physically manipulate host cell machinery to perpetuate their genomes at the host's expense. In addition to hijacking host macromolecular complexes to make new viral products, viruses are known to modulate the host response to infection in order to escape detection and prevent the host from interfering with viral replication [1]–[3]. Many viral proteins directly compete with host proteins for binding sites [4], and some even modify host proteins chemically, *e.g.* marking them for degradation by the host's own machinery [5]–[7]. Despite providing such detailed information about the molecular mechanisms and consequences of specific exogenous interactions, traditional virology studies are highly focused and thus are often unable to draw general conclusions about the mechanisms governing exogenous interactions even among closely related viruses. As a result, despite these detailed studies on specific host-virus interaction systems, the overarching principles that distinguish host-virus interactions from within-host interactions have not yet been elucidated.

A systems biology approach is therefore essential in order to obtain a global perspective on host-pathogen interactions. Recent advances in high-throughput experimental and computational biology have enabled the reconstruction and analysis of large-scale host-pathogen PPI networks [8]–[18]. These systematic studies have successfully revealed global patterns in host-pathogen systems that are otherwise inaccessible by the traditional reductionist approach, which studies host-pathogen PPIs one at a time. For example, global analyses have revealed that viral proteins have repeatedly evolved to target host proteins central to the host PPI network (*e.g.* hubs with many physical interaction partners) [8], [11], [19]. In addition to targeting common host pathways regulating viral infection and replication in general [11], [20], different classes of viruses also target host pathways uniquely involved in class-specific mechanisms of infection and replication [20]. Despite these advances, current host-pathogen systems biology approaches are highly abstract and coarse-grained, treating proteins as nodes and PPIs as edges; therefore, the insights generated by these analyses are strongly limited in spatial and mechanistic resolution. A high-resolution approach is needed to uncover more general rules governing host-pathogen PPI networks [21].

One approach to increase resolution in PPI networks has been to construct three-dimensional (3D) structural models to protein interactions [22]–[25]. We recently applied this technique to build an atomic-resolution map of human-virus and within-human PPI networks by constructing 3D structural models of exogenous and endogenous PPIs using homology modeling [4]. A direct comparison between the resulting human-virus and within-human structural interaction networks revealed systematic and significant differences between exogenous and endogenous interactions that are otherwise hidden in the binary PPI networks. For example, we found that viral proteins preferentially bind to and mimic existing endogenous interfaces on their human target proteins, rather than creating entirely new interfaces. In addition, interface mimicry tends to be achieved without structural similarity in the human-virus PPI network as compared to the within-human PPI network. Finally, endogenous interfaces mimicked by virus proteins tend to evolve quickly, and mediate many endogenous interactions that are transient and regulatory in function, as compared to generic endogenous interfaces [4]. Although 3D structure information can be used to interrogate host-pathogen interaction networks at atomic resolution in a reasonably unbiased manner, coverage in these analyses is limited by the number of high-quality 3D homology models that can be built for endogenous and exogenous interactions [26].

**Figure 1:**
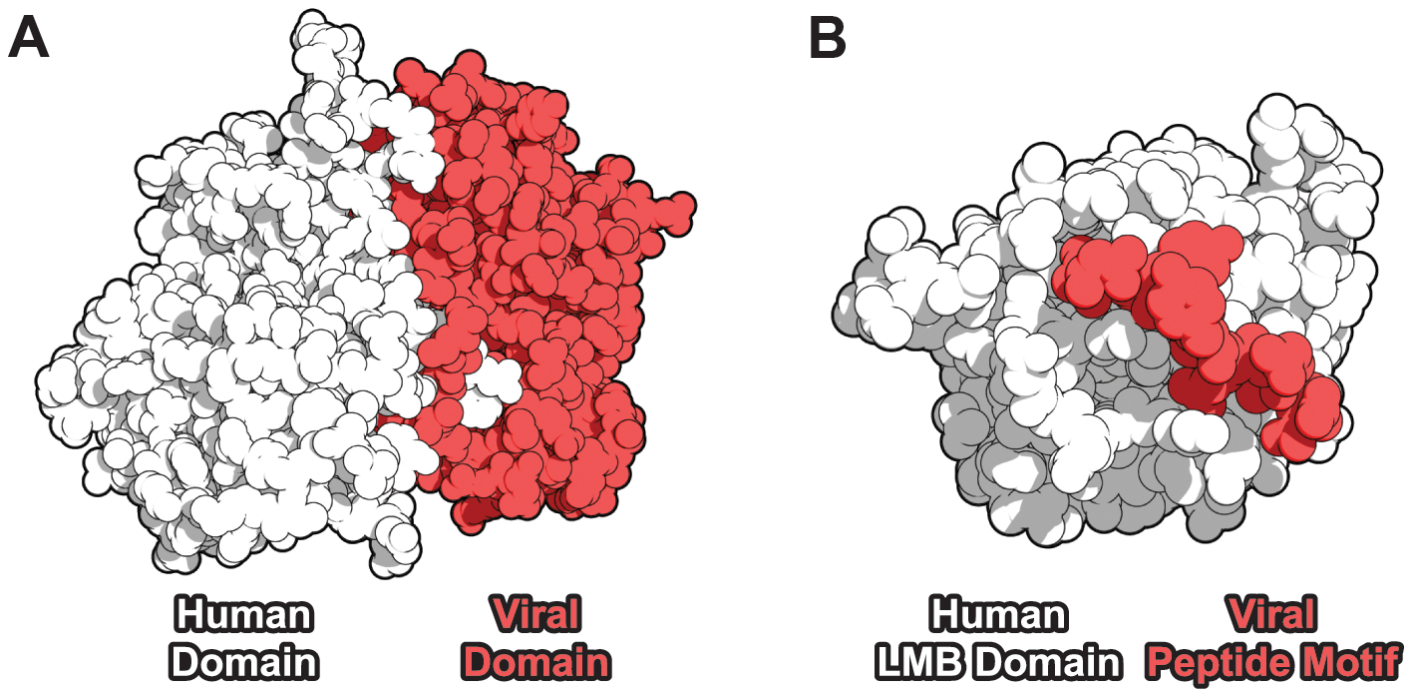
Domain-centric mechanisms of host-virus protein-protein interaction. (**A**) A domain-domain interaction (DDI) example [43]: a cyclin domain-containing protein from Saimiriine herpesvirus 2 (red) targets a human CDK6 kinase domain (white). (**B**) A domain-motif interaction (DMI) example [44]: human retinoblastoma-associated protein (white) contains a linear motif-binding (LMB) domain which recognizes the peptide motif LxCxE (red) in the human papillomavirus E7 protein.

In this work, we probe high-resolution principles governing exogenous and endogenous PPI networks using a domain-resolved approach that annotates proteins with known domains, and PPIs with known domain-centric interaction mechanisms (domain-domain interactions and domain-motif interactions; Figure 1). This domain-resolved network is of higher accuracy than the binary PPI network, and of higher coverage than the 3D structural interaction network. Although domain-based studies of host-pathogen PPIs have been previously reported for specific pathogens [27], [28], a systematic, quantitative comparison between exogenous and endogenous PPI networks at the level of domains has never been attempted before. Domain-motif interactions have been previously reported to be important in host-pathogen interactions [29], but their prevalence in the global host-pathogen interaction network remains unknown relative to the within-host network [29]. Our global, domain-resolved map of human-virus and within-human PPI networks enables, for the first time, the discovery of novel systematic and statistically significant differences between exogenous and endogenous PPIs in terms of domain interaction usage. While two human proteins competing to bind the same domain tend to have global sequence similarity, viral proteins competing with human proteins do not. Viral proteins preferentially target human proteins containing linear motif-binding domains independent of their degree in the endogenous network. In addition, viral proteins use linear motifs to mediate protein-protein interactions more often than human proteins do. Finally, viral proteins contain a higher density of linear motifs than generic human proteins. Collectively, these observations suggest that the exogenous network is very different from the endogenous network in terms of domain interaction usage. While the endogenous network evolves largely by gene duplication followed by divergence, the exogenous network is dominated by convergent evolution of domain-motif and domain-domain interactions. Compared to human proteins, viral proteins tend to convergently evolve and pack multiple linear motifs mediating many biophysical interactions that are functionally diverse in order to manipulate complex host processes. Together, these results strongly support the utility of a domain-resolved approach for interrogating host-pathogen interaction networks, and in particular for determining the general principles that distinguish exogenous and endogenous interactions.

## Results

### A high-coverage, high-accuracy domain-resolved human-virus interaction network

We constructed high-resolution human-virus (*exogenous*) and within-human (*endogenous*) protein-protein interaction (PPI) networks by annotating proteins and PPIs with known domain information. We considered two major categories of domain-centric interaction mechanisms: domain-domain interactions (DDIs), in which a globular domain from one protein binds to a globular domain from a second protein (Figure 1A), and domain-motif interactions (DMIs), in which a linear motif-binding (LMB) domain from one protein binds to a short, linear peptide motif in a second protein (Figure 1B). Some PPIs can be annotated with both DDI and DMI mechanisms.

**Figure 2:**
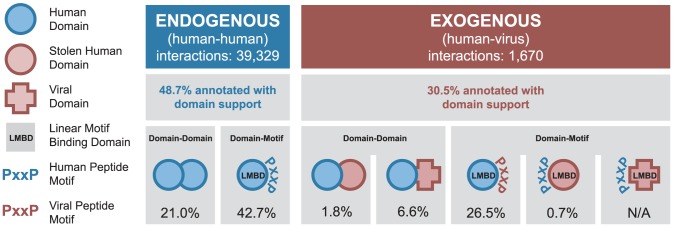
Coverage of human-virus protein-protein interaction network by domain-centric interaction mechanisms. Fractions of endogenous and exogenous PPIs that can be assigned to different domain-centric interaction mechanisms (DDIs and DMIs). Each mechanism is illustrated using the symbols at the left, with the percentage of interactions described by that mechanism given below. An interaction may be described by more than one interaction mechanism.

The endogenous portion of our network contains 39,329 PPIs among 9,870 human proteins, of which 48.7% can be assigned to at least one of the two domain-centric mechanisms (Figure 2). There are 8,277 DDIs mediated by 1,164 unique domain types forming 3,209 unique types of interacting domain pairs. In addition, there are 16,785 DMIs mediated by 554 unique LMB domain types (Figure 2).

The exogenous portion of our network contains 1,670 interactions between 267 viral proteins and their 954 human protein targets. The viral proteins represent 17 viral families and 7 Baltimore classes (Table S1) [30]. 30.5% of all exogenous interactions can be assigned at least one domain-centric interaction mechanism, which can be further divided into the following five cases (Figure 2). (i) 30 exogenous DDIs involve a human domain homolog present in a viral protein, presumably due to horizontal gene transfer (Table S2). (ii) 110 exogenous DDIs involve human domains interacting with virus-specific domains. (iii) 443 exogenous DMIs are mediated by an LMB domain-containing human protein binding to a viral protein with the corresponding linear motif. (iv) 11 exogenous DMIs are mediated by a viral protein containing a human-like LMB domain binding to a human protein with the corresponding linear motif (e.g. the SH2 and SH3 domain-containing Src protein from Avian sarcoma virus, and the kinase domain-containing BGLF4 protein from Epstein-Barr virus). (v) Exogenous DMIs mediated by virus-specific LMB domains are only just starting to be characterized [31], and are not represented in our network.

### Domain-resolved interactions are of high quality

**Figure 3:**
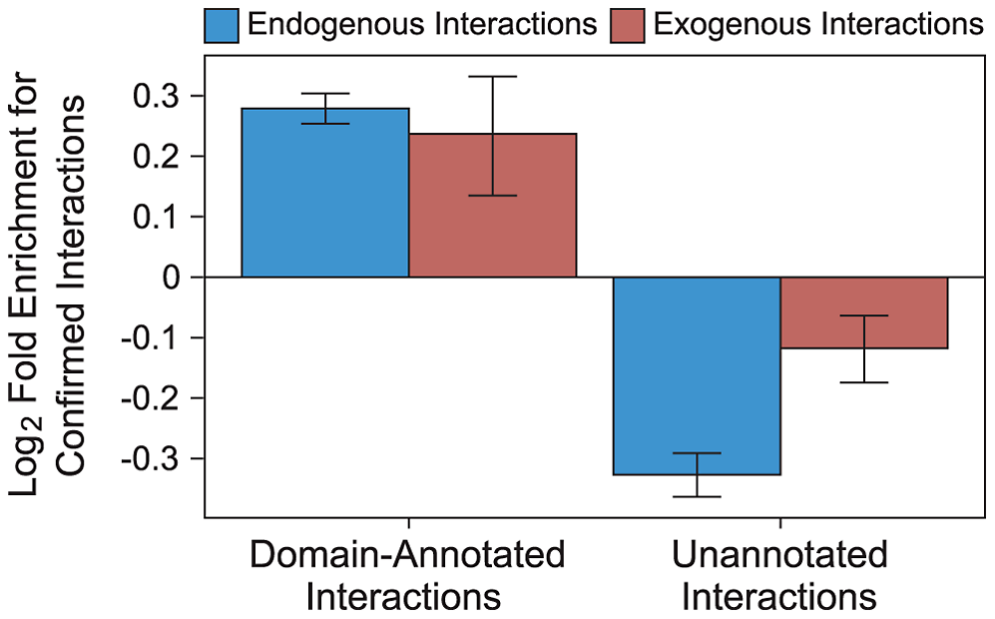
Domain-resolved interactions are of high quality. Endogenous (blue) and exogenous (red) PPIs that are supported by a known DDI or DMI are enriched for confirmed interactions (*i.e.*, interactions reported by at least two publications). Endogenous and exogenous PPIs lacking the support of domain-centric mechanisms are depleted for confirmed interactions. Error bars reflect the standard error based on 1,000 rounds of bootstrap resampling.

Annotating exogenous and endogenous PPI networks with domain-centric interaction mechanisms yields networks with increased resolution compared to binary networks, while maintaining high coverage. We annotated proteins with complete taxonomic and Pfam domain information [32], [33], and PPIs with interacting domain information [34]–[36]. These annotated PPIs are of higher quality than generic PPIs, as measured by the overlap with a gold standard set of PPIs reported by at least two independent publications (“confirmed interactions”). Specifically, endogenous interactions annotated with domain-centric mechanisms are 52% more likely to be confirmed than non-annotated endogenous interactions (Figure 3), and exogenous interactions annotated with domain-centric mechanisms are 28% more likely to be confirmed than non-annotated exogenous interactions (Figure 3). Hence, in addition to providing mechanistic insights, annotation of endogenous and exogenous interactions with domain interaction information raises our confidence in the accuracy of the underlying interactions.

### Binding site mimicry evolves differently in virus and host proteins

In our previous work based on 3D structural models of exogenous and endogenous interactions, we demonstrated that viral proteins frequently bind to human target proteins at sites of existing endogenous interfaces (“interface mimicry”) [4]. Moreover, compared to overlap among endogenous interfaces, exogenous-endogenous interface overlap was much less likely to involve global structural similarity between the two proteins targeting the same interface [4]. Here, we reexamined this result in the context of our domain-resolved human-virus PPI network.

**Figure 4:**
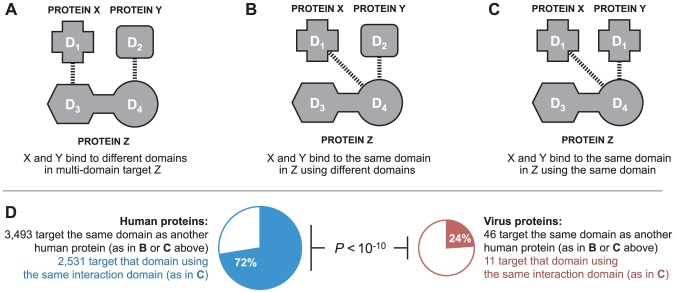
Binding site mimicry evolves differently in virus and host proteins. Two proteins can participate in DDIs with a common target by binding to: (**A**) different domains in the target; (**B**) the same domain in the target using different interaction domains; or, (**C**) the same domain in the target using the same interaction domain. (**D**) Viral proteins are significantly less likely than human proteins to bind to the same domain of a human protein by means of domain sequence similarity to an endogenous binding partner (Fisher's exact test, two-tailed *P*<10^−10^).

In the absence of 3D structural information, it is not possible to determine if two proteins bind to the same interface on a third protein. However, in our domain-resolved human-virus PPI network, it is possible to determine if two proteins bind to the same domain in the third protein (Figure 4A–C), which is a prerequisite for interface mimicry. A similar approach has been previously used in the yeast 3D structural interaction network to distinguish between singlish-interface hub proteins, which mediate mutually exclusive PPIs, and multi-interface hub proteins, which mediate multiple simultaneous PPIs [22]. Among DDIs in the endogenous network, of the 3,493 cases where two human proteins bind to the same domain of a third human protein, 72% are mediated by domains sharing significant sequence similarity (Figure 4D). In contrast, among DDIs in the combined exogenous-endogenous network, of the 46 cases where a viral protein and a human protein bind to the same domain of another human protein, only 24% are mediated by domains sharing significant sequence similarity (Figure 4D). The results from these domain-resolved analyses are consistent with our previous findings using 3D structural networks: viral proteins are significantly less likely than human proteins to bind to the same domain of a human target protein by means of global sequence similarity to an endogenous binding partner (Fisher's exact test, two-tailed *P*<10^−10^; Figure 4D).

### Viruses tend to target LMB domain-containing human proteins

**Figure 5:**
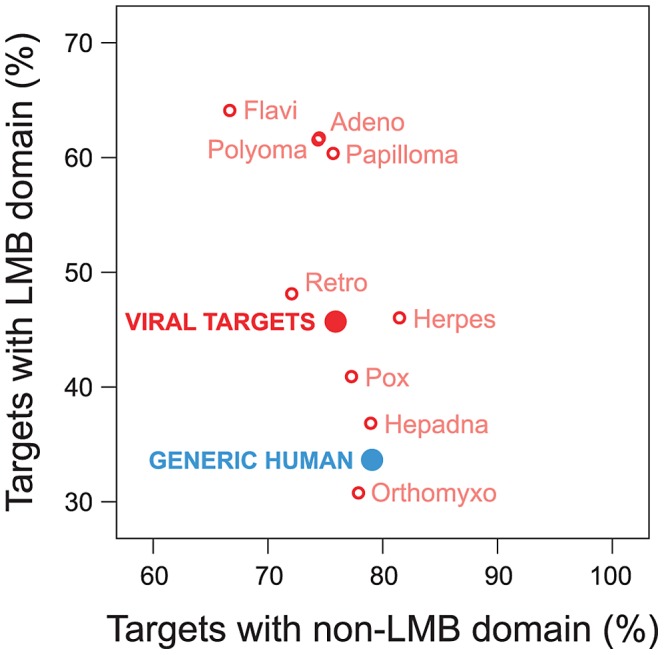
Viruses tend to target human proteins containing linear motif-binding (LMB) domains. We compared domain composition of generic human proteins in the endogenous network (blue) to human proteins targeted by viruses (“viral targets”, red). The vertical axis indicates the fraction of proteins in a group containing LMB domains; the horizontal axis indicates the fraction of proteins containing non-LMB domains. Relative to generic human proteins, human proteins targeted by viruses are significantly more likely to contain an LMB domain (vertical axis; Fisher's exact test, two-tailed *P*<10^−15^), and are slightly less likely to contain non-LMB domains (horizontal axis; *P* = 0.012).

Viruses have been known to use linear peptide motifs to target endogenous LMB domains [21], [29]; however, it is unknown how prevalent this mechanism of interaction is. Here, we quantified how frequently viral proteins target host proteins using a domain-motif interaction mechanism. We examined the domain composition of human proteins targeted by viruses, and compared it with the domain composition of generic human proteins in the network. We found that human proteins targeted by viruses are significantly enriched for LMB domains relative to generic human proteins (Fold enrichment = 1.36; Fisher's exact test, two-tailed *P*<10^−15^; Figure 5). With the exception of Orthomyxoviruses, the direction of this trend holds for exogenous interactions from all major viral families in the network, and cannot be attributed to a specific type of virus (Figure 5). In contrast, the difference in enrichment for non-LMB domains between human proteins targeted by viruses and generic human proteins is only marginally significant (Fold enrichment = 0.96; *P* = 0.012; Figure 5), suggesting that the observed enrichment for LMB domains among human proteins targeted by viruses is not a simple result of superior domain annotation among these proteins.

### Preferential targeting of LMB domains by viruses is independent of host protein degree

Previous work has revealed a tendency for viral proteins to target host protein hubs [11], [27], [28]. Because LMB domains recognize small peptide motifs which may occur in many proteins, we expect LMB domain-containing human proteins to participate in more endogenous interactions than proteins without LMB domains, and hence be more hub-like. Indeed, the average LMB domain-containing human protein in our network participates in 10.5 endogenous interactions, while the average LMB domain-free protein participates in only 6.4 endogenous interactions. As a result, our finding that viruses tend to target LMB domain-containing proteins may be confounded by the viral preference for targeting hub proteins.

**Figure 6:**
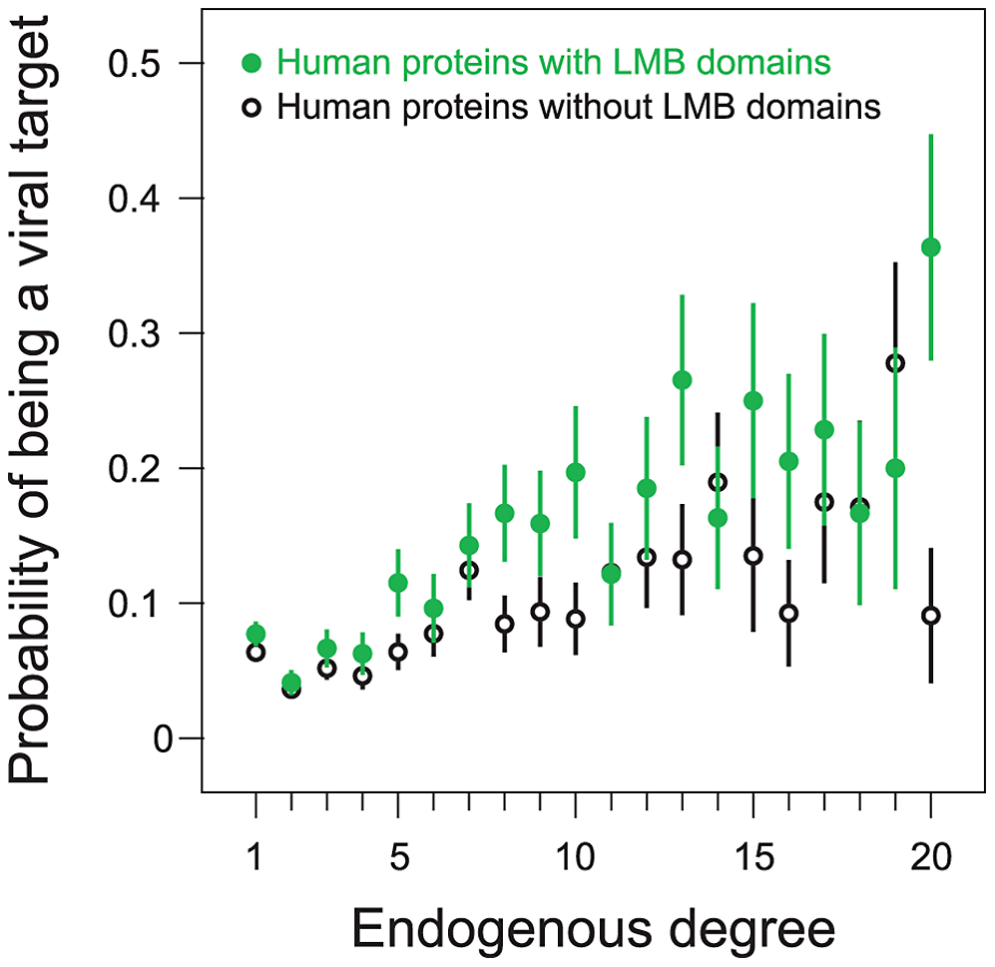
Preferential targeting of LMB domains by viruses is independent of host protein degree. We partitioned human proteins into LMB domain-containing proteins (filled green points), and LMB domain-free proteins (open black points). We further divided proteins according to endogenous degree. 92% of all human proteins in the network fall into one of the degree 1–20 bins; each bin contains at least 20 proteins. The probability of being a viral target increases with degree for both LMB domain-containing and LMB domain-free proteins. For a given degree, LMB domain-containing proteins are more likely to be viral targets than LMB domain-free proteins.

We examined the effects of endogenous degree on the relationship between a human protein containing an LMB domain and the likelihood of that protein being a viral target. We stratified human proteins according to endogenous degree and then compared the probability of being a viral target among proteins with and without LMB domains (Figure 6). Consistent with previous findings that viruses target host protein hubs, we observe that the probability of being a viral target increases with increasing endogenous degree, and that this trend holds for both LMB domain-containing proteins and LMB domain-free proteins (Figure 6). More importantly, for a fixed endogenous degree, LMB domain-containing human proteins are more likely to be targeted by viruses than human proteins without LMB domains (Figure 6). This finding suggests that viruses preferentially target LMB domain-containing human proteins independent of their higher average degree.

To quantify the statistical significance of this assertion, we measured concordance between (i) having an LMB domain and (ii) being a viral target, among pairs of human proteins with the same degree. We picked a pair of proteins with the same degree in which one had an LMB domain while the other did not, and considered the pair *concordant* if the LMB domain-containing protein was a viral target whereas the LMB domain-free protein was not, and *discordant* if the LMB domain-containing protein was not a viral target whereas the LMB domain-free protein was. We observed a strong preference for concordant protein pairs over discordant protein pairs (58% concordant versus 42% discordant), favoring a degree-independent association between LMB domain-containing proteins and viral targets. The degree-independent association between a human protein containing an LMB domain and being a viral target is statistically significant (one-tailed *P* = 0.006; Figure 6), as calculated by a degree-preserving random permutation of LMB domain and viral target annotations among sets of human proteins.

### Viral proteins have a higher fraction of domain-motif interactions than human proteins

The results of the previous section establish that viruses tend to preferentially target human proteins containing LMB domains by comparing properties of human proteins targeted by viruses against all other human proteins. Next, we determined if the viral preference for targeting LMB domain-containing proteins also holds at the level of PPIs, when comparing the fraction of domain-motif interactions (DMIs) between viral proteins and human proteins.

**Figure 7:**
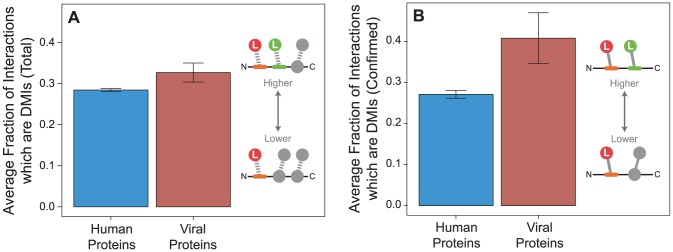
Viral proteins have a higher fraction of domain-motif interactions (DMIs) than human proteins. Fraction of DMIs out of the total number of interactions per protein tend to be higher in viral proteins (red) than human proteins (blue) in (**A**) all interactions in the network (permutation test, two-tailed *P* = 0.047), and (**B**) confirmed interactions (*P* = 0.018). Error bars reflect the standard error.

We observed that viral proteins have higher fraction of DMIs out of total number of PPIs per protein than human proteins (permutation test, two-tailed *P* = 0.047; Figure 7A). To ensure this trend was not due to superior annotation in either the endogenous or exogenous dataset, we repeated the analyses on confirmed interactions and observed the same trend (*P* = 0.018; Figure 7B). This result suggests that although the endogenous network contains more proteins and PPIs and has a higher fraction of domain annotation than the exogenous network (Figure 2), viral proteins are more likely on average than human proteins to interact using a domain-motif interface (Figure 7).

### Viral proteins target LMB domains at greater density than human proteins

We next examined whether viral preference for targeting LMB domain-containing proteins is reflected in elevated linear motif occurrence in viral proteins as compared to human proteins. We determined density of linear motifs and LMB domains targeted per protein, rather than directly comparing the total number of linear motifs and LMB domains targeted per protein, to account for the large difference in protein size between viral and human proteins: within our network, the median viral protein length (306 residues) is 34% smaller than the median human protein length (464 residues).

**Figure 8:**
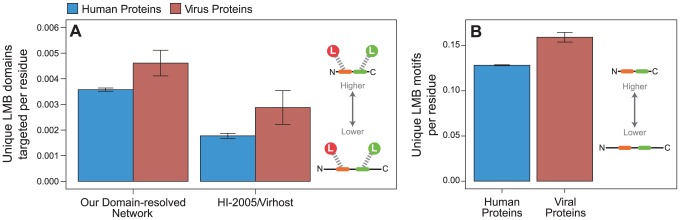
Viral proteins target LMB domains at greater density than human proteins. Viral proteins (red) target significantly more unique LMB domains per residue than human proteins (blue) in (**A**) all interactions in the network (permutation test, two-tailed *P* = 0.012), and an independently generated human-virus PPI network [37], [38] (*P* = 0.049). (**B**) Viral proteins also have significantly more unique LMB motif types per residue than human proteins (*P*<0.001). Error bars reflect the standard error.

We first calculated the density of unique LMB domains targeted per residue for viral proteins and human proteins. We found that viral proteins target a greater variety of unique LMB domains per residue than human proteins (permutation test, two-tailed *P* = 0.012; Figure 8A). This calculation directly compares the properties of experimentally determined endogenous and exogenous PPIs, and may be confounded by methodological differences in mapping endogenous versus exogenous interactions: only 22% of endogenous interactions are reported by small-scale experiments (reporting fewer than 100 interactions), whereas as many as 73% of exogenous interactions are reported by small-scale experiments. To ensure that the aforementioned trend observed in our network cannot be explained by this difference in methodology, we repeated the analyses on a host-virus PPI network built from the previously published “HI-2005” and “VirHost” interactome datasets, which were generated using the same methodology [37], [38], and observed the same trend (*P* = 0.049; Figure 8A). This analysis supports our earlier conclusion that viral proteins interact with a greater variety of distinct LMB domains on a per residue basis than human proteins.

Our observation that viral proteins target more LMB domains per residue than human proteins may still be confounded by subtle differences in experimental procedures for mapping endogenous versus exogenous interactomes. To control for such differences, we calculated the density of linear motif types per residue for each viral and human protein, regardless of whether the motifs were used to mediate known interactions. This measure is interactome-independent, and thus is free of any procedural biases in experimental interactome maps. Consistent with our previous findings, we found that viral proteins have significantly more unique linear motif types per residue than human proteins (*P*<0.001; Figure 8B). These results indicate that in addition to preferentially targeting LMB domain-containing proteins (Figures 5 and 6), viral proteins are more likely to target a greater variety of unique LMB domains per residue than human proteins (Figure 7), and have a higher density of unique linear motifs than human proteins (Figure 8).

## Discussion

We constructed a domain-resolved map of host-virus and within-host protein-protein interaction (PPI) networks to probe general, systems-level principles that distinguish host-pathogen, exogenous PPIs from within-host, endogenous PPIs. Annotation of proteins and interactions with known domain information yields a domain-resolved network with higher resolution and quality than the binary PPI network, and higher coverage than the 3D structural interaction network. Classification of endogenous and exogenous PPIs into domain-domain interactions (DDIs) and domain-motif interactions (DMIs) reveals global differences in domain interaction patterns between host-pathogen and within-host networks that are otherwise hidden in traditional binary PPI networks. While our domain-centric annotations reduce the rate of false positives in PPI networks, additional potential limitations include false negatives, incomplete annotation, and methodological biases. In this work, we have minimized the effects of such incompleteness and biases by carefully controlling for them when performing systematic comparisons between exogenous and endogenous networks. A potentially more significant limitation is investigator bias: most host-pathogen studies are conducted on clinically significant human pathogens, such as HIV. Despite this investigator bias, our exogenous network represents a wide variety of viral families (Table S1). We emphasize that our comparisons and contrasts between exogenous and endogenous PPIs are carried out within our domain-resolved interaction networks, and therefore our conclusions should be minimally confounded by systematic biases inherent in a domain-resolved approach.

Our analyses reveal systematic, mechanistic differences between exogenous and endogenous interactions. The most pronounced of these differences is the tendency for viruses to mimic human interactions by means of convergent evolution. We find that viral proteins and human proteins tend to target the same domain of another human protein without any shared sequence similarity, extending the results of our previous work using 3D structural interaction networks [4]. In addition, we demonstrate for the first time that viral proteins are more likely than human proteins to mediate interactions using short linear motifs, which can easily arise by convergent evolution due to their small size and minimal genomic constraints. These observations support the hypothesis that viral proteins tend to convergently evolve mechanisms to mimic existing endogenous binding interfaces. In addition, viral proteins are more economical and functionally more pleiotropic than human proteins in that viral proteins target more LMB domain-containing proteins, and also target more unique LMB domains per residue. Furthermore, we found that viral proteins contain more unique linear motif types per residue. Given the knowledge that linear motifs in disordered regions tend to be conserved and are more likely to be the target of binding by LMB domains [39], we further investigated whether or not viral proteins are more disordered than human proteins. Indeed, we find that viral proteins are enriched for disorder-promoting residues [40] relative to human proteins (Student's *t*-test, two-tailed *P*<0.0001). Additionally, considering only motifs in “disordered regions” (a region ±10 residues around a motif, containing >60% disorder-promoting residues [40]), we observe that viral proteins continue to have higher density of unique linear motif types per residue than human proteins do (permutation test, two-tailed *P*<0.05).

Our results demonstrate that viral proteins and virus-host PPIs are in general very different from host proteins and within-host PPIs: viral proteins are small, complex, multifunctional polypeptides which can mediate multiple host-virus interactions, typically using the highly economical and highly pleiotropic method of domain-motif interactions largely through convergent evolution. These signatures of pleiotropy, economy, and convergent evolution in the virus-host PPI network are a direct consequence of the intense selection pressure on viruses to establish and maintain, with very limited genomic resources at their disposal, extensive and effective physical interactions with the host necessary for their survival. These global trends are applicable in general to viral proteins and exogenous interactions, and do not reflect a bias in a specific viral type, nor in a specific methodology for determining PPIs. Our results suggest that annotating viral proteins with domain-centric interaction mechanisms, especially by scanning viral protein sequences for linear motifs, can provide a novel approach to identifying host protein interaction partners for study. It may also be possible to use this domain-centric annotation approach to identify therapeutic treatments based on competition for motif binding sites. Thus, our study highlights the importance of a high-resolution, domain-resolved approach to host-pathogen network biology for revealing general mechanistic principles governing host-pathogen interactions.

## Methods

### Assembling endogenous and exogenous protein-protein interaction data

We collected reports of endogenous (human-human) protein-protein interactions (PPIs) from the IntAct database, and reports of exogenous (human-virus) PPIs from the IntAct and VirusMINT databases [41], [42]. We discarded PPIs with missing protein sequence information in UniProt [33]. Exogenous PPIs were further filtered to exclude (i) virus species that do not normally target mammalian hosts, and (ii) deltaviruses, which (as subviral satellites) cannot infect a host without co-infection by another virus. The viral proteins represent 17 viral families and all Baltimore classes [30].

### Annotating protein-protein interactions with domains and interaction mechanisms

We assigned Pfam domains to the human and viral proteins in our networks using the Pfam batch search utility, subject to an *E*-value cutoff of 10^−2^ [32]. To avoid misclassifying proviral fragments in the human proteome as native human domains, we removed human proteins from the analysis if they were annotated as viral fragments or polyproteins in Uniprot. Using protein sequence and domain information, we then assigned putative interaction mechanisms to endogenous and exogenous PPIs in our dataset. We classified PPIs as domain-domain interactions (DDIs) if a domain in the first protein was known or predicted to interact with a domain in the second protein. Pairs of putative interacting domains were assembled from the DOMINE database [36], which integrates results from a variety of DDI curation and prediction studies. In addition, we classified PPIs as domain-motif interactions (DMIs) if one of the proteins contained a putative linear motif-binding (LMB) domain and the second protein contained a linear motif recognized by that LMB domain. We utilized predicted domain-motif associations from Neduva *et al.* [35] and manually curated domain-motif associations from the database of Eukaryotic Linear Motifs (ELM) [34]. Motifs in these datasets take the form of regular expressions which can be searched directly against an amino acid sequence using standard pattern matching tools.

### Comparison of datasets and statistical analyses

To assess the quality of the endogenous and exogenous networks, we compared them individually against a gold standard set of endogenous and exogenous PPIs; these subsets of interactions were constructed by querying for interactions that were reported by at least two independent publications.

We measured concordance between having an LMB domain and being a viral target by picking pairs of human proteins with the same degree in which one had an LMB domain while the other did not. The protein pair was *concordant* if the LMB domain-containing protein was a viral target and the LMB domain-free protein was not a viral target. Conversely, the protein pair was *discordant* if the LMB domain-containing protein was not a viral target and the LMB domain-free protein was a viral target. All other protein pairs were considered to be non-informative. To evaluate the statistical significance of this test, we completed 1,000 repetitions of random permutation of the LMB domain and viral target annotations among sets of human proteins with the same endogenous degree and repeated our procedure.

For permutation-based comparisons of virus and human proteins, we first compute the mean of each group and then evaluate the difference between these means. To evaluate if such a difference is likely to arise at random, we repeatedly permute the “virus” and “human” protein labels and then calculate the difference in the means of the newly randomized groups. Over a large number of trials (*e.g.* 1000), the fraction of permutations in which the random difference is at least as large as the observed difference approximates the probability of observing such a difference at random (*p*-value), and serves as a measure of the statistical significance of the observed measurement.

## Supporting Information

Table S1 This table lists the number of unique proteins and total number of interactions reported for each viral family found in the network, as annotated by the 2011 release of the International Committee on Taxonomy of Viruses.
(XLSX)

Table S2 This table lists the 17 unique human domains that are observed in viral proteins to mediate 30 domain-domain interactions with human proteins.
(XLSX)

Table S3 This table lists the endogenous domain-resolved PPIs. Included in the table are UniProt IDs, protein descriptions, and species annotation for the interacting protein pair. We also provide the classification of domain-centric interaction mechanism: (1) a human protein with a human domain binding to a second human protein with a human domain (“Human Domain : Human Domain”); and, (2) a human protein containing a human linear motif-binding domain, binding to a human protein containing the corresponding motif (“(Human Domain : Motif) OR (Motif : Human Domain)”). Interacting domains or domain-motif pairs are separated by a colon (:). If a protein has more than one pair of interacting domains or domain-motifs, they are separated by a vertical bar (|). Domains are listed by their Pfam annotation ID, and motifs are listed as a searchable regular expression.
(XLSX)

Table S4 This table lists the exogenous domain-resolved PPIs. Proteins are listed with human proteins followed by viral proteins, with UniProt ID, protein description, and species annotation for each protein. We have included the classification of domain-centric interaction mechanism: (1) a human protein with a human domain binding to a viral protein with a human domain (“Human Domain : Human Domain”); (2) a human protein with a human domain binding to a viral protein with a viral domain (“Human Domain : Virus Domain”); (3) a human protein containing a human linear motif-binding domain, binding to a viral protein containing the corresponding motif (“Human Linear Motif-Binding Domain : Virus Motif”); and, (4) a viral protein containing a human linear motif binding domain, binding to a human protein with the corresponding motif (“Human Motif : Human Linear Motif-Binding Domain”). Interacting domains or domain-motif pairs are separated by a colon (:). If a protein has more than one pair of interacting domains or domain-motifs, they are separated by a vertical bar (|). Domains are listed by their Pfam annotation ID, and motifs are listed as a searchable regular expression.
(XLSX)
